# Identification of RNA biomarkers for chemical safety screening in mouse embryonic stem cells using RNA deep sequencing analysis

**DOI:** 10.1371/journal.pone.0182032

**Published:** 2017-07-27

**Authors:** Hidenori Tani, Jun-ichi Takeshita, Hiroshi Aoki, Kaoru Nakamura, Ryosuke Abe, Akinobu Toyoda, Yasunori Endo, Sadaaki Miyamoto, Masashi Gamo, Hiroaki Sato, Masaki Torimura

**Affiliations:** 1 Environmental Management Research Institute, National Institute of Advanced Industrial Science and Technology (AIST), Onogawa, Tsukuba, Ibaraki, Japan; 2 Research Institute of Science for Safety and Sustainability, National Institute of Advanced Industrial Science and Technology (AIST), Onogawa, Tsukuba, Ibaraki, Japan; 3 College of Engineering Systems, School of Science and Engineering, University of Tsukuba, Tennodai, Tsukuba, Ibaraki, Japan; 4 Department of Risk Engineering, Faculty of Systems and Information Engineering, University of Tsukuba, Tennodai, Tsukuba, Ibaraki, Japan; 5 Research Institute for Sustainable Chemistry, National Institute of Advanced Industrial Science and Technology (AIST), Higashi, Tsukuba, Ibaraki, Japan; University of Texas at Austin Dell Medical School, UNITED STATES

## Abstract

Although it is not yet possible to replace in vivo animal testing completely, the need for a more efficient method for toxicity testing, such as an in vitro cell-based assay, has been widely acknowledged. Previous studies have focused on mRNAs as biomarkers; however, recent studies have revealed that non-coding RNAs (ncRNAs) are also efficient novel biomarkers for toxicity testing. Here, we used deep sequencing analysis (RNA-seq) to identify novel RNA biomarkers, including ncRNAs, that exhibited a substantial response to general chemical toxicity from nine chemicals, and to benzene toxicity specifically. The nine chemicals are listed in the Japan Pollutant Release and Transfer Register as class I designated chemical substances. We used undifferentiated mouse embryonic stem cells (mESCs) as a simplified cell-based toxicity assay. RNA-seq revealed that many mRNAs and ncRNAs responded substantially to the chemical compounds in mESCs. This finding indicates that ncRNAs can be used as novel RNA biomarkers for chemical safety screening.

## Introduction

The 7th Amendment to the Cosmetics Directive banned animal testing of cosmetic ingredients for human use in 2013 [1]. Although it is not yet possible to replace in vivo animal testing completely, the need for a more efficient method for toxicity testing has been widely acknowledged [2]. Among the alternative methods to animal testing, the use of in vitro cell-based assays appears to be one of the most appropriate approaches to predict the toxic properties of single chemicals, particulate matter, complex mixtures and environmental pollutants [3–9].

Over the past decade, global gene expression profiling has been used increasingly to investigate cellular toxicity in transformed and primary cells [6]. Almost all previous studies used transformed cells such as Jurkat [10], A549 [5], or HepG2 cells [7,8], or primary cells such as human pulmonary artery endothelial cells [11], human bronchial epithelial cells [12], or human aortic endothelial cells [13].

These previous studies only focused on mRNAs as biomarkers. However, recent studies identified non-coding RNAs (ncRNAs) as efficient novel biomarkers for toxicity testing [14–16]. ncRNAs can be roughly classified into three groups: small ncRNAs (20‒30 nucleotides [nt]) such as microRNAs (miRNAs), intermediate-sized ncRNAs (30‒200 nt) such as small nucleolar RNAs (snoRNAs), and long ncRNAs (lncRNAs; >200 nt) such as long intergenic non-coding RNAs (lincRNAs). LncRNAs are defined as RNA molecules greater than 200 nucleotides in length that do not contain any apparent protein-coding potential [17–20]. The majority of lncRNAs are transcribed by RNA polymerase II (Pol II), as evidenced by Pol II occupancy, 5′ caps, histone modifications associated with Pol II transcriptional elongation, and polyadenylation. Moreover, the previous studies used transformed or primary cells. Transformed cells are genetically altered, typically aneuploid, and may exhibit clinically irrelevant toxic responses to compounds. Primary cells from animal tissues lose their in vivo phenotypes, can exhibit high variability among isolations, and can often only be expanded by dedifferentiation [21].

The present study used deep sequencing analysis (RNA-seq) to identify novel RNA biomarkers including ncRNAs that exhibited substantial responses to general chemical toxicity from nine chemicals, and to benzene toxicity specifically. The nine chemicals are listed in the Japan Pollutant Release and Transfer Register as class I designated chemical substances. Moreover, we used mouse embryonic stem cells (mESCs) because mESCs have three important attributes [9,22]: (i) normality: they are regarded as native cells; (ii) pluripotency, the ability to differentiate into specialized cells; and (iii) self-renewal, the ability to undergo numerous cycles of cell division while remaining undifferentiated in culture. These characteristics make mESCs a promising choice for assessment of toxicity, and overcome the limitations of transformed or primary cells.

## Materials and methods

### Chemicals

Benzene, bis(2-ethylhexyl)phthalate, chloroform, p-cresol, p-dichlorobenzene, phenol, pyrocatechol, tri-n-butyl phosphate, and trichloroethylene were obtained from Wako, Japan. These chemicals were dissolved in dimethyl sulfoxide (DMSO) (Wako) and diluted in culture medium at 0.1% vol/vol final concentration.

### Cell culture

The H-1 mESC line was originally isolated from C3H/He mice [23]. mESCs were maintained in Dulbecco’s modified Eagle’s medium (4.5 g/l glucose) with L-glutamine, without sodium pyruvate, (Nacalai Tesque, Japan) supplemented with 15% foetal bovine serum (Gibco, USA), 1000 U/ml Stem Sure Leukemia Inhibitory Factor (mouse recombinant solution; Wako), 0.1 mM Stem Sure 2-mercaptoethanol solution (Wako), and penicillin–streptomycin (Gibco). Cells were grown on mitomycin C (Kyowa Kirin, Japan)-treated mouse embryonic fibroblast feeder cells (C57BL/6J) at 37°C in a humidified incubator with 5% CO_2_. For chemical stress treatments, mESCs were cultured in ESGRO Complete Plus serum-free clonal grade medium (Merck Millipore, Germany) on gelatine (Sigma, USA)-coated dishes without feeder cells.

### Chemical stress treatments

Cells were seeded at 3.8 × 10^5^ cells per well of a 6-well plate in 2 ml medium. The cells were incubated overnight at 37°C with 5% CO_2_. In separate analyses, cells were treated with benzene (final concentration 1000 μM) and bis(2-ethylhexyl)phthalate (100 μM), chloroform (1000 μM), p-cresol (10 μM), p-dichlorobenzene (100 μM), phenol (100 μM), pyrocatechol (10 μM), tri-n-butyl phosphate (10 μM), or trichloroethylene (1000 μM) for 24 h. Total RNA was extracted from cells in the 6-well plates with RNAiso Plus (Takara, Japan) according to the manufacturer’s instructions.

### RNA-seq and data analysis

RNA-seq analyses were performed by Takara. Ribosomal RNA was removed using a Ribo-Zero Magnetic Gold kit (Human/Mouse/Rat; Illumina, USA). An RNA-seq library was constructed using a TruSeq Standard mRNA Sample Prep kit (Illumina). One hundred base paired-end read RNA-seq tags were generated using an Illumina HiSeq 2500 sequencer according to the standard protocol. The fluorescence images were processed to sequences using the analysis pipeline supplied by Illumina. RNA-seq tags were mapped to the mouse genome (hg19) from the National Center for Biotechnology Information using TopHat mapping software. More than 40 million RNA-seq tags from each sample were analysed. Genic representations using fragments per kilobase of exon per million mapped fragments (FPKM) to normalize for gene length and depth of sequencing were calculated. Sequencing tags were then mapped to the mouse reference genome sequence using mapping software, allowing no mismatches. RNA-seq tags were assigned to corresponding RefSeq transcripts when their genomic coordinates overlapped. We used RNA sequences available from public databases: mRNA from NM of RefSeq and lncRNA candidates from NR of RefSeq [24]. In total, 32,586 RNAs from the NM and NR categories of the RefSeq Database were used for RNA annotation. The following expression ratio r (x, y) was used in this study.

$$r(x,y)={log}_{2}\frac{y}{x},$$

where x and y were the FPKMs of the control and treatment groups, respectively. Note that if x and y were zero, then the smallest values (excluding zero) in the control and the treatment groups were used instead of x and y, respectively.

### Real-time quantitative reverse-transcription polymerase chain reaction (RT-qPCR)

Total RNA was extracted from cells with RNAiso Plus (TaKaRa) according to the manufacturer’s instructions. The isolated RNA was reverse transcribed into cDNA using PrimeScript RT Master Mix (Perfect Real Time; TaKaRa). The resulting cDNA was amplified using the following primer sets: Gapdh (forward: 5’-CCGGGAAACTGTGGCGTGATGG-3’, reverse: 5’-AGGTGGAGGAGTGGGTGTCGCTGTT-3’); NM_001177607 (forward: 5’-GCTGTGGAGTTGCTGCCTA-3’, reverse: 5’-AGGAGAGGAGAGGAGCATCA-3’), NM_178734 (forward: 5’-GGAAAGCCTTTGCTCAGAGA-3’, reverse: 5’-CATAGGGCTTCTCCCCAGT-3’); NR_027375 (forward: 5’-TGATTTGACTTTGCTTCATAGGG-3’, reverse: 5’-TGAATCGAACCATTTTGTACTGA-3’); NM_001166648 (forward: 5’-ACTCTGTTCAAGAAAAAGGGTTGT-3’, reverse: 5’-TCCATGAAAAGTTCAGCCATT-3’); NM_001163553 (forward: 5’-AAAGCTGCTCCTTGTGTCTCA-3’, reverse: 5’-AAGGCCAAAGACCTAGCACA-3’); NM_145978 (forward: 5’-GCTGCTCACCACTTGACCTA-3’, reverse: 5’-ATGGAGCAGCACCCTCACT-3’). Gapdh was used for normalization. THUNDERBIRD SYBR qPCR mix (Toyobo, Japan) was used according to the manufacturer’s instructions. RT-qPCR analysis was performed using a MyiQ2 (BIO-RAD, USA).

### Data access

Short-read sequence archive data in this study are registered in GenBank (http://www.ncbi.nlm.nih.gov/genbank)/DDBJ (http://ddbj.sakura.ne.jp). The data used to determine the expression levels of transcripts are registered as accession numbers DRX076650‒DRX076669.

## Results

### General up- and downregulation of mRNAs and ncRNAs after chemical exposure

**Fig 1:**
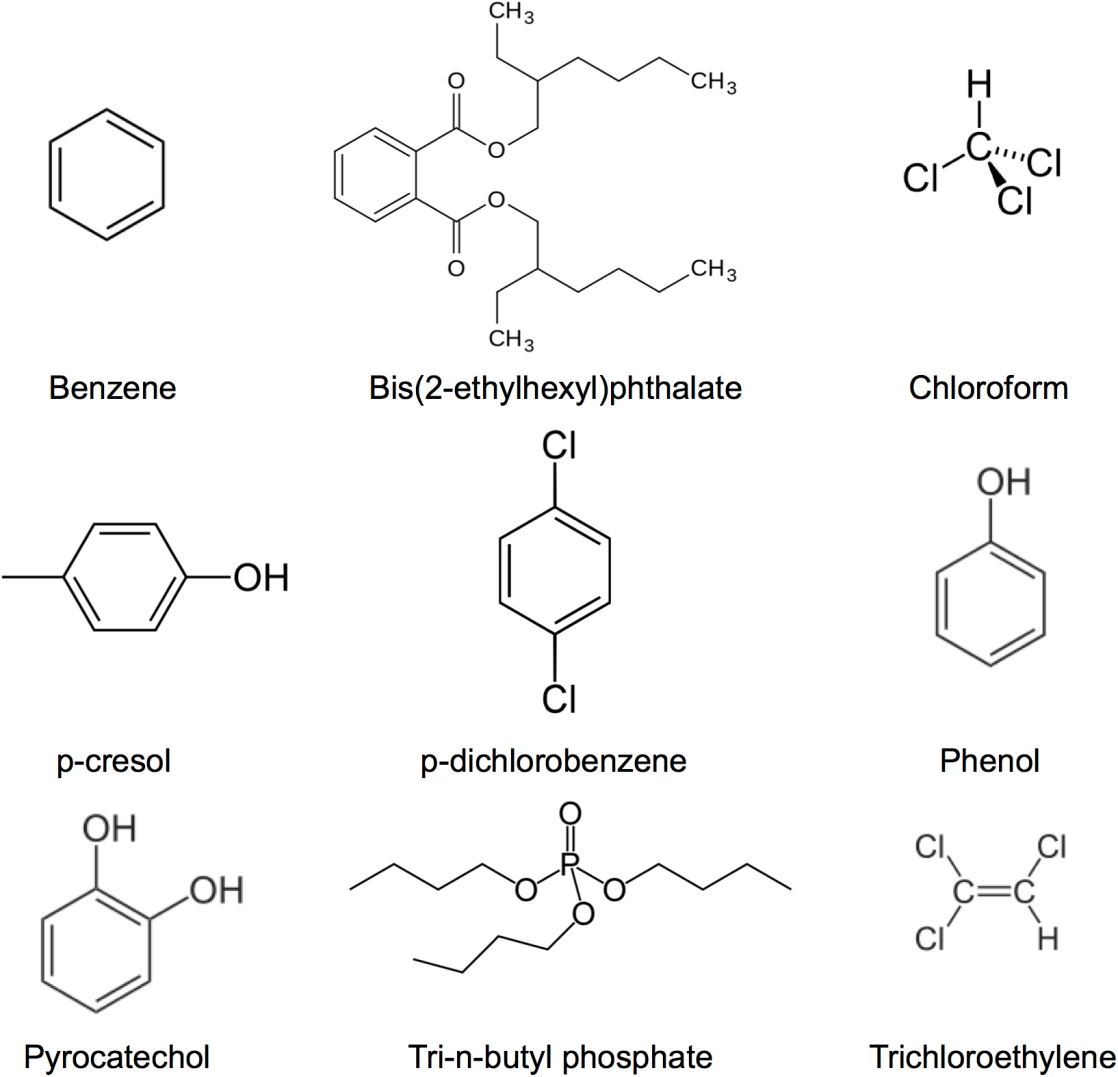
Chemical structures used in the present study.

mESCs were exposed to nine chemicals [benzene, bis(2-ethylhexyl)phthalate, chloroform, p-cresol, p-dichlorobenzene, phenol, pyrocatechol, tri-n-butyl phosphate, and trichloroethylene] (Fig 1) for 24 hours in duplicate. In preliminary experiments, we optimized the concentrations of chemicals as described previously [16]. We identified the 30 RNAs whose expression was most upregulated following the exposure of mESCs to the nine chemicals in general (Table 1). We found that mRNA levels for these genes increased by approximately 100- to 30,000-fold after exposure to the chemicals. To confirm the reproducibility of the RNA-seq data, we determined the RNA expression levels by RT-qPCR in duplicate for Top 3 for upregulation of mRNAs and ncRNAs after benzene exposures. The results showed that the relative quantitative values (exposure/control) of NM_001177607, NM_178734, and NR_027375 were 746.6 ± 96.1, 570.3 ± 150.6, and 606.4 ± 52.4 (mean ± errors), respectively. The data of RT-qPCR were similar to those of RNA-seq. Thus, we confirmed the reproducibility of the RNA-seq data. We then categorized the upregulated mRNAs according to their Gene Ontology (GO) terms (Table 2). Of the various GO terms, genes for regulation of cellular responses, such as cellular response to mechanical stimulus, cellular response to reactive oxygen species, and negative regulation of inflammatory response, occurred particularly frequently among the upregulated genes. Moreover, two ncRNAs, NR_027375 (Ythdf3_v3) and NR_033430 (Gm2694) were identified as being upregulated by general chemical exposure. The lengths of Ythdf3_v3 and Gm2694 are 5,308 nt and 682 nt, respectively. The functions of these ncRNAs are unknown; therefore, we cannot perform the correspondence analysis between ncRNA and the expression of mRNA.

**Table 1:** Genes upregulated in mouse embryonic stem cells on general exposure to nine chemicals (Top 30).

Refseq	Exposure/Control								
	benzene	bis(2-ethyl hexyl) phthalate	chloroform	p-cresol	p-dichloro benzene	phenol	pyrocatechol	tri-n-butyl phosphate	trichloro ethylene
NM_001177607	2667	10637	3034	4930	14728	3121	4024	4676	8851
NM_178734	6834	3616	3499	5184	6391	2972	10310	5005	3287
NR_027375	4830	2395	6836	6228	4882	8832	4264	5970	7685
NM_172778	1815	4058	3750	4191	4393	3768	3655	3802	2614
NM_177574	4950	1747	3613	4149	3466	1835	3924	4718	2599
NM_013512	3874	2151	2892	5323	1922	3904	7376	2713	1403
NM_001285498	2527	6516	9953	3416	5001	2618	5952	2911	8049
NM_133879	131	5965	8323	7471	8088	6402	5370	6160	3259
NM_001276493	5927	3976	6333	1380	1199	7397	600	11589	6744
NM_145382	6295	2543	5371	6476	3558	14154	1939	4669	9346
NM_145382	3775	3095	2735	3653	2784	1681	439	4915	4993
NM_001253736	4538	6404	3700	4562	4994	4928	4680	2756	3753
NM_025946	1372	3026	946	1504	2090	1422	1644	794	1804
NM_178045	3542	2905	1077	1992	3147	5589	4233	538	1644
NM_153501	861	1397	4525	10424	527	3024	6467	2069	3225
NM_001164745	12322	8244	11169	35385	11178	12894	14437	10698	10323
NM_207232	201	1726	3008	1086	695	1594	2526	3292	1305
NM_025674	798	9090	4841	3148	878	2974	5633	306	13590
NM_001024922	3422	2344	3642	4357	1851	3617	1503	4806	1948
NM_001082536	1749	2416	3389	4744	534	3912	3696	5762	2549
NR_033430	5262	1814	952	1600	1491	1347	1680	2009	1375
NM_198620	3886	4663	3861	5967	3962	3577	2409	6865	8069
NM_001193660	2783	5634	4021	7849	13592	4257	3003	2417	8249
NM_146142	1500	1293	3280	1177	474	2813	2232	1134	1014
NM_001136079	1176	2035	2585	2151	3214	2266	2955	1320	3541
NM_145483	506	756	801	402	755	393	740	897	594
NM_001113181	2284	2804	3197	1385	3439	1324	1024	2693	2534
NM_001289471	2082	1819	1032	877	1083	1497	926	1178	1923
NM_001048179	2638	470	329	1605	615	1686	3883	2109	1169
NM_027062	2758	1797	3139	2482	858	2932	2014	1736	1363

**Table 2:** GO terms for genes upregulated in mouse embryonic stem cells on general exposure to nine chemicals (Top 30).

RefSeq	GO term	Definition
NM_001177607	0003677	DNA binding
NM_178734	0006355	regulation of transcription, DNA-templated
NR_027375	-	-
NM_172778	0016491	oxidoreductase activity
NM_177574	0015031	protein transport
NM_013512	0008092	cytoskeletal protein binding
NM_001285498	0071300	cellular response to retinoic acid
NM_133879	0006355	regulation of transcription, DNA-templated
NM_001276493	0071260	cellular response to mechanical stimulus
NM_145382	0005737	cytoplasm
NM_145151	0003677	regulation of transcription, DNA-templated
NM_001253736	0008270	zinc ion binding
NM_025946	0034614	cellular response to reactive oxygen species
NM_178045	0007049	cell cycle
NM_153501	0019217	regulation of fatty acid metabolic process
NM_001164745	0016791	phosphatase activity
NM_207232	0016311	dephosphorylation
NM_025674	0010468	regulation of gene expression
NM_001024922	0010501	RNA secondary structure unwinding
NM_001082536	0045893	positive regulation of transcription, DNA-templated
NR_033430	-	-
NM_198620	0005575	cellular_component
NM_001193660	0005198	structural molecule activity
NM_146142	0030154	cell differentiation
NM_001136079	0050728	negative regulation of inflammatory response
NM_145483	0006355	regulation of transcription, DNA-templated
NM_001113181	0006811	ion transport
NM_001289471	0006355	regulation of transcription, DNA-templated
NM_001048179	0071677	positive regulation of mononuclear cell migration
NM_027062	0002376	immune system process

Next, we identified the 30 RNAs whose expression was most downregulated following the exposure of mESCs to the nine chemicals in general (Table 3). We found that the mRNA levels for these genes decreased to approximately 0.0001- to 0.006 times their original levels after exposure to the chemicals. To confirm the reproducibility of the RNA-seq data, we determined the RNA expression levels by RT-qPCR in duplicate for Top 3 for downregulation of mRNAs after benzene exposures. The results showed that the relative quantitative values (exposure/control) of NM_001166648, NM_001163553, and NM_145978 were 0.0006 ± 0.0001, 0.0003 ± 0.0002, and 0.0007 ± 0.0002 (mean ± errors), respectively. The data of RT-qPCR were similar to those of RNA-seq. Thus, we confirmed the reproducibility of the RNA-seq data. We then categorized the downregulated mRNAs according to their GO terms (Table 4). Of the various GO terms, genes for regulation of cellular processes, such as regulation of transcription, negative regulation of apoptosis, and regulation of cellular metabolism, occurred particularly frequently among the downregulated genes. Moreover, five ncRNAs, NR_040383 (4930520O04Rik), NR_033540 (F630042J09Rik), NR_121603 (Atp11a_v4), NR_102360 (Zbtb24_v4), and NR_105027 (1700124L16Rik) were identified as being downregulated by chemical exposure. The lengths of 4930520O04Rik, F630042J09Rik, Atp11a_v4, Zbtb24_v4, and 1700124L16Rik are 1,217 nt, 3,154 nt, 7,648 nt, 2,872 nt, and 346 nt, respectively. The functions of these ncRNAs are unknown; therefore, we cannot perform the correspondence analysis between ncRNA and the expression of mRNA.

**Table 3:** Genes downregulated in mouse embryonic stem cells on general exposure to nine chemicals (Top 30).

Refseq	Exposure/Control								
	benzene	bis(2-ethyl hexyl) phthalate	chloroform	p-cresol	p-dichloro benzene	phenol	pyrocatechol	tri-n-butyl phosphate	trichloro ethylene
NM_001166648	0.00070	0.00017	0.00022	0.00017	0.00017	0.00017	0.00017	0.00022	0.00017
NM_001163553	0.00021	0.00021	0.00021	0.00042	0.00018	0.00021	0.00021	0.00024	0.00021
NM_145978	0.00026	0.00035	0.00026	0.00026	0.00026	0.00033	0.00025	0.00026	0.00025
NR_040383	0.00048	0.00048	0.00065	0.00048	0.00048	0.00048	0.00048	0.00048	0.00048
NM_001285431	0.00024	0.00024	0.00015	0.00024	0.00039	0.00024	0.00024	0.00024	0.00015
NM_146228	0.00051	0.00051	0.00051	0.00051	0.00051	0.00051	0.00051	0.00061	0.00483
NM_001164420	0.00079	0.00079	0.00079	0.00079	0.00079	0.00079	0.00079	0.00079	0.00074
NM_001291009	0.00079	0.00079	0.00079	0.00079	0.00079	0.00049	0.00079	0.00079	0.00079
NM_001163336	0.00050	0.00049	0.00050	0.00069	0.00050	0.03919	0.00049	0.00050	0.00049
NR_033540	0.00070	0.00070	0.00510	0.00070	0.00071	0.00070	0.00070	0.00068	0.00109
NM_178061	0.00038	0.00038	0.00213	0.00009	0.00284	0.00165	0.00117	0.00218	0.00082
NM_146248	0.00014	0.00016	0.00014	0.00016	0.00166	0.00016	0.00225	0.00081	0.00014
NM_021302	0.00089	0.00075	0.00075	0.00075	0.00075	0.00075	0.00063	0.00075	0.00075
NM_001081362	0.00066	0.00024	0.00066	0.00040	0.00040	0.00040	0.00040	0.00024	0.00039
NM_001145968	0.00063	0.00069	0.00069	0.00069	0.00083	0.00069	0.00069	0.00069	0.00075
NR_121603	0.00092	0.00091	0.00092	0.00121	0.00092	0.00092	0.00092	0.00121	0.00091
NM_030178	0.00039	0.00038	0.00129	0.00025	0.00039	0.00039	0.00059	0.00025	0.00025
NR_102360	0.00022	0.00038	0.00022	0.00022	0.00038	0.00038	0.00021	0.00012	0.00012
NM_001285875	0.00090	0.00099	0.00091	0.00123	0.00091	0.00091	0.00082	0.00091	0.00090
NM_145598	0.00063	0.00063	0.00089	0.00063	0.00089	0.00063	0.00063	0.00045	0.00063
NM_021374	0.00151	0.00150	0.00151	0.00151	0.00186	0.00151	0.00150	0.00151	0.00150
NM_207201	0.00079	0.00079	0.00097	0.00079	0.00079	0.00096	0.00079	0.01820	0.00079
NM_175938	0.00070	0.00083	0.00070	0.00065	0.00070	0.00075	0.00092	0.00075	0.00069
NM_001162921	0.00068	0.00068	0.00068	0.00137	0.00068	0.00068	0.00137	0.00092	0.03173
NM_018812	0.00079	0.00010	0.00481	0.00010	0.00013	0.00018	0.00013	0.00620	0.00010
NM_146188	0.00031	0.00021	0.00026	0.00031	0.00026	0.00026	0.00021	0.00031	0.00026
NR_105027	0.00036	0.00036	0.00051	0.00036	0.00051	0.00051	0.00051	0.00072	0.00051
NM_001242378	0.00262	0.00261	0.00263	0.00262	0.00263	0.00263	0.00261	0.00263	0.00261
NM_001271542	0.00035	0.00047	0.00048	0.00032	0.00048	0.00071	0.00047	0.00071	0.00047
NM_009400	0.00055	0.00054	0.00099	0.00054	0.00055	0.00030	0.00030	0.00055	0.00099

**Table 4:** GO terms for genes upregulated in mouse embryonic stem cells exposed to benzene (Top 30).

RefSeq	GO term	Definition
NM_001166648	0006355	regulation of transcription, DNA-templated	
NM_001163553	0006886	intracellular protein transport	
NM_145978	0046872	metal ion binding	
NR_040383	-	-	
NM_001285431	0006355	regulation of transcription, DNA-templated	
NM_146228	0005096	GTPase activator activity	
NM_001164420	0005575	cellular_component	
NM_001291009	0043433	negative regulation of sequence-specific DNA binding transcription factor activity	
NM_001163336	0070588	calcium ion transmembrane transport	
NR_033540	-	-	
NM_178061	0046872	metal ion binding	
NM_146248	0030154	cell differentiation	
NM_021302	0016310	phosphorylation	
NM_001081362	0006355	regulation of transcription, DNA-templated	
NM_001145968	0005575	cellular_component	
NR_121603	-	-	
NM_030178	0045893	positive regulation of transcription, DNA-templated	
NR_102360	-	-	
NM_001285875	0043066	negative regulation of apoptotic process	
NM_145598	0045494	photoreceptor cell maintenance	
NM_021374	0009968	negative regulation of signal transduction	
NM_207201	0007186	G-protein coupled receptor signaling pathway	
NM_175938	0031324	negative regulation of cellular metabolic process	
NM_001162921	0004519	endonuclease activity	
NM_018812	0006355	regulation of transcription, DNA-templated	
NM_146188	0007275	multicellular organism development	
NR_105027	-	-	
NM_001242378	0030641	regulation of cellular pH	
NM_001271542	0010629	negative regulation of gene expression	
NM_009400	0043066	negative regulation of apoptotic process	

### Specific up- and downregulation of mRNAs and ncRNAs after exposure to benzene

We next explored toxic response to specific chemical exposure, using benzene as a representative chemical substance. We identified the 30 RNAs whose expression was most upregulated following the exposure of mESCs to benzene (Table 5). We found that mRNA levels for these genes increased by approximately 3000- to 13,000-fold after exposure to benzene. We then categorized the upregulated mRNAs according to their GO terms (Table 5). Of the various GO terms, genes involved in cellular responses, such as cellular response to mechanical stimulus, inflammatory response, and cellular response to DNA damage, occurred particularly frequently among the upregulated genes. Moreover, two ncRNAs, NR_038062 (Yipf2_v4) and NR_027375 (Ythdf3_v3) were identified as being upregulated by exposure to benzene. The lengths of Yipf2_v4 and Ythdf3_v3 are 1,919 nt and 5,306 nt, respectively. The functions of these ncRNAs are unknown; therefore, we cannot perform the correspondence analysis between ncRNA and the expression of mRNA.

**Table 5:** Genes upregulated in mouse embryonic stem cells exposed to benzene (Top 30).

RefSeq	Exposure/Control	GO term	Definition
NM_001193619	13838	0043066	negative regulation of apoptotic process
NM_001164745	12322	0016791	phosphatase activity
NR_038062	10355	-	-
NM_001004185	9065	0007050	cell cycle arrest
NM_133992	9012	0006397	mRNA processing
NM_001102611	7884	0032259	methylation
NM_029132	6994	0042802	identical protein binding
NM_178734	6834	0006355	regulation of transcription, DNA-templated
NM_001177710	6465	0030100	regulation of endocytosis
NM_145382	6295	0005737	cytoplasm
NM_001287015	5977	0000398	mRNA splicing, via spliceosome
NM_001276493	5927	0071260	cellular response to mechanical stimulus
NM_001166413	5831	0035023	regulation of Rho protein signal transduction
NM_134161	5412	0016740	ransferase activity
NM_001048008	5250	0005515	protein binding
NM_028081	5065	0006355	regulation of transcription, DNA-templated
NM_001163702	5021	0004842	ubiquitin-protein transferase activity
NM_177574	4950	0015031	protein transport
NR_027375	4830	-	-
NM_029612	4782	0005575	cellular_component
NM_001253870	4765	0070062	extracellular exosome
NM_001253736	4538	0070062	extracellular exosome
NM_001164735	4356	0006954	inflammatory response
NM_001159714	4230	0008380	RNA splicing
NM_001301641	4094	0006915	apoptotic process
NM_001145957	4034	0003674	molecular_function
NM_198620	3886	0008150	biological_process
NM_013512	3874	0005856	cytoskeleton
NM_145151	3775	0006355	regulation of transcription, DNA-templated
NM_009685	3766	0006355	cellular response to DNA damage stimulus

Next, we identified the 30 RNAs whose expression was most downregulated following the exposure of mESCs to benzene (Table 6). We found that mRNA levels for these genes decreased to approximately 0.000002 to 0.0002 times their original levels after exposure to benzene. We then categorized the downregulated mRNAs according to their GO terms (Table 6). Of the various GO terms, genes involved in regulation of cellular processes, such as multicellular organism development, cell cycle, and DNA replication, occurred particularly frequently among the downregulated genes. Moreover, two ncRNAs, NR_034050 (Snora44) and NR_102360 (Zbtb24_v4) were identified as being downregulated by exposure to benzene. The lengths of Snora44 and Zbtb24_v4 are 117 nt and 2,872 nt, respectively. Snora44 is a snoRNA. The functions of these ncRNAs are unknown; therefore, we cannot perform the correspondence analysis between ncRNA and the expression of mRNA. Other chemical compound exposure data are shown in S1–S16 Tables.

**Table 6:** Genes downregulated in mouse embryonic stem cells exposed to benzene (Top 30).

RefSeq	Exposure/Control	GO term	Definition
NR_034050	0.0000023	-	-
NM_026489	0.0000582	0051321	meiotic cell cycle
NM_011158	0.0001113	0045859	regulation of protein kinase activity
NM_001080118	0.0001137	0071383	cellular response to steroid hormone stimulus
NM_001033528	0.0001196	0006511	ubiquitin-dependent protein catabolic process
NM_001159571	0.0001266	0007155	cell adhesion
NM_001289839	0.0001307	0006355	regulation of transcription, DNA-templated
NM_001168516	0.0001338	0016740	transferase activity
NM_001081373	0.0001338	0007049	cell cycle
NM_146248	0.0001358	0007275	multicellular organism development
NM_153796	0.0001362	0006260	DNA replication
NM_007554	0.0001379	0030509	BMP signaling pathway
NM_172252	0.0001406	0003723	RNA binding
NM_181424	0.0001469	0044065	regulation of respiratory system process
NM_001159498	0.0001498	0050687	negative regulation of defense response to virus
NM_001289726	0.0001514	0007275	multicellular organism development
NM_001256522	0.0001643	0008284	positive regulation of cell proliferation
NM_181423	0.0001682	2000827	mitochondrial RNA surveillance
NM_177352	0.0001732	0006071	glycerol metabolic process
NM_013846	0.0001738	0007275	multicellular organism development
NM_028705	0.0001908	0005829	cytosol
NM_001077364	0.0001928		regulation of transcription, DNA-templated
NM_172409	0.0001968	0016043	cellular component organization
NM_001164185	0.0002027	0019216	regulation of lipid metabolic process
NM_001163553	0.0002083	0006886	intracellular protein transport
NM_026303	0.0002109	0008152	metabolic process
NM_029811	0.0002136	0070374	positive regulation of ERK1 and ERK2 cascade
NR_102360	0.0002153	-	-
NM_009538	0.0002200	0010468	regulation of gene expression

## Discussion

In this study, we used RNA-seq to identify novel RNA biomarkers that exhibited a substantial response to general chemical toxicity from nine chemicals, and to benzene toxicity specifically. Some ncRNAs exhibited substantial responses to the chemical compounds, although fewer ncRNAs than mRNAs responded in this way. We considered that both mRNAs and ncRNAs expression levels might be independently changed by chemical stresses. We identified two ncRNAs (Ythdf3_v3 and Gm2694) that were upregulated and five ncRNAs (4930520O04Rik, F630042J09Rik, Atp11a_v4, Zbtb24_v4 and 1700124L16Rik) that were downregulated in response to general chemical exposure. These results indicate that ncRNAs as well as mRNAs have the potential to be surrogate indicators of chemical safety screening. We also identified two ncRNAs (Yipf2_v4 and Ythdf3_v3) that were upregulated and two ncRNAs (Snora44 and Zbtb24_v4) that were downregulated in benzene-treated cells. These findings indicate that ncRNAs can be used as novel RNA biomarkers for chemical safety screening.

Traditional RNA biomarkers of various types of cell stress have been identified, for example markers of oxidative stress response (Nfkb1, Jun, and Hif1a), DNA damage (Ppp1r15a, Gadd45a, Ddit3, and Cdkn1a), heat shock response (Hsp90aa1 and Hsf1), and endoplasmic reticulum stress (Atf3 and Bbc3), and hypoxia inducible factors (Arnt and Mtf1) [25]. However, the expression levels of these RNA biomarkers did not appear among the 30 genes that were the most up- or downregulated by chemical exposure in this study. Therefore, we identified novel RNA biomarkers that were more efficient markers of chemical toxicity than traditional RNA biomarkers.

As expected, we observed upregulation of genes involved in regulation of cellular responses when cells were treated with the nine chemicals in general. This result suggests that the cells responded to the stress by increasing expression of genes involved in cellular responses. A similar phenomenon was observed in cells treated with benzene. Furthermore, we observed downregulation of genes involved in regulation of cellular processes when cells were treated with the nine chemicals in general. This suggests that cells downregulated basic processes such as proliferation in response to the cellular stress by decreasing expression of genes involved in these cellular processes.

Profiles for small RNAs such as miRNAs have been reported for several animal species including humans, mice, and rats [26–30]. miRNAs play pivotal roles in regulation of gene expression, and have the potential to be useful biomarkers. However, small RNAs and long RNAs cannot be analysed at the same time using RNA-seq because they require different RNA-seq application systems. lncRNAs have great potential to be useful biomarkers; for example, lncRNAs participate in diverse cellular functions including chromatin modification, transcription, splicing, mRNA decay, translation, and protein transport and assembly, and their RNA elements and RNA-protein complex machineries are also thought to be extremely diverse. We therefore focused on lncRNAs in the present study. Moreover, mESCs can differentiate into a variety of cell types [31], and thus allow assessment of chemical exposure risk in a variety of tissues and cell types. However, in the present study we used undifferentiated mESCs because we aimed to provide a basic framework for using mESCs for chemical safety screening.

We propose that many mRNAs and ncRNAs represent novel RNA biomarkers for chemical safety screening using mESCs. This study provides only a basic framework for such an application, and we plan to assess differentiated cells derived from mESCs, such as neurons, cardiomyocytes, and hepatocytes. We believe that these potential RNA biomarkers will be used for chemical safety screening in the future. For example, they could be quantified by a custom-made microchip or array [32].

## Supporting information

S1 Table Specific up-regulated genes in mouse embryonic stem cells exposed to bis(2-ethylhexyl)phthalate (Top 30). (PDF)

S2 Table Specific up-regulated genes in mouse embryonic stem cells exposed to chloroform (Top 30). (PDF)

S3 Table Specific up-regulated genes in mouse embryonic stem cells exposed to p-cresol (Top 30). (PDF)

S4 Table Specific up-regulated genes in mouse embryonic stem cells exposed to p-dichlorobenzene (Top 30). (PDF)

S5 Table Specific up-regulated genes in mouse embryonic stem cells exposed to phenol (Top 30). (PDF)

S6 Table Specific up-regulated genes in mouse embryonic stem cells exposed to pyrocatechol (Top 30). (PDF)

S7 Table Specific up-regulated genes in mouse embryonic stem cells exposed to tri-n-butyl phosphate (Top 30). (PDF)

S8 Table Specific up-regulated genes in mouse embryonic stem cells exposed to trichloroethylene (Top 30). (PDF)

S9 Table Specific down-regulated genes in mouse embryonic stem cells exposed to bis(2-ethylhexyl)phthalate (Top 30). (PDF)

S10 Table Specific down-regulated genes in mouse embryonic stem cells exposed to chloroform (Top 30). (PDF)

S11 Table Specific down-regulated genes in mouse embryonic stem cells exposed to p-cresol (Top 30). (PDF)

S12 Table Specific down-regulated genes in mouse embryonic stem cells exposed to p-dichlorobenzene (Top 30). (PDF)

S13 Table Specific down-regulated genes in mouse embryonic stem cells exposed to phenol (Top 30). (PDF)

S14 Table Specific down-regulated genes in mouse embryonic stem cells exposed to pyrocatechol (Top 30). (PDF)

S15 Table Specific down-regulated genes in mouse embryonic stem cells exposed to tri-n-butyl phosphate (Top 30). (PDF)

S16 Table Specific down-regulated genes in mouse embryonic stem cells exposed to trichloroethylene (Top 30). (PDF)
