# Supplementary material for: Identification of RNA biomarkers for chemical safety screening in mouse embryonic stem cells using RNA deep sequencing analysis
Source: PLoS One. 2017 Jul 27;12(7):e0182032. doi: 10.1371/journal.pone.0182032 (PMC5531504; doi:10.1371/journal.pone.0182032)
Supplement: S1 Table — (PDF) [file pone.0182032.s001.pdf]

S1 Table. Specific up-regulated genes in mouse embryonic stem cells exposed to bis(2-ethylhexyl)phthalate (Top 30)

| Refseq | Exposure/Control |
|---|---|
| NM_001243968 | 13829 |
| NM_001177607 | 10637 |
| NM_001162973 | 9627 |
| NM_025674 | 9090 |
| NM_001252520 | 8516 |
| NM_001285498 | 6516 |
| NM_133879 | 5965 |
| NM_001243132 | 5206 |
| NM_001301305 | 5188 |
| NM_001083329 | 5050 |
| NM_153423 | 4829 |
| NM_001008501 | 4666 |
| NM_001198831 | 4330 |
| NM_001290807 | 4221 |
| NM_001168679 | 4209 |
| NM_139297 | 4198 |
| NM_015814 | 4160 |
| NM_172778 | 4058 |
| NM_001276493 | 3976 |
| NR_110343 | 3974 |
| NM_001276485 | 3969 |
| NM_001286540 | 3875 |
| NM_009601 | 3805 |
| NM_001282064 | 3771 |
| NM_001290148 | 3756 |
| NM_178734 | 3616 |
| NM_007465 | 3502 |
| NM_029612 | 3327 |
| NM_198884 | 3246 |
| NM_001099624 | 3242 |
