# Supplementary material for: Identification of RNA biomarkers for chemical safety screening in mouse embryonic stem cells using RNA deep sequencing analysis
Source: PLoS One. 2017 Jul 27;12(7):e0182032. doi: 10.1371/journal.pone.0182032 (PMC5531504; doi:10.1371/journal.pone.0182032)
Supplement: S2 Table — (PDF) [file pone.0182032.s002.pdf]

S2 Table. Specific up-regulated genes in mouse embryonic stem cells exposed to chloroform (Top 30)

Refseq	Exposure/Control
NM_025669	75340
NM_001291482	16210
NM_001164745	11169
NM_001285498	9953
NM_153792	9612
NM_001276455	9564
NM_007395	9418
NM_133879	8323
NM_134161	7493
NM_198884	7455
NM_001291818	7086
NR_027375	6836
NM_001042592	6405
NM_001276493	6333
NM_009952	5826
NM_001166413	5576
NM_181585	5523
NM_145382	5371
NM_032396	5228
NM_011997	5017
NM_025674	4841
NM_011629	4769
NM_001004185	4707
NM_001013368	4625
NM_153501	4525
NM_145415	4382
NM_001083329	4366
NM_001163640	4159
NM_001102468	4060
NM_001193660	4021
