# Supplementary material for: Identification of RNA biomarkers for chemical safety screening in mouse embryonic stem cells using RNA deep sequencing analysis
Source: PLoS One. 2017 Jul 27;12(7):e0182032. doi: 10.1371/journal.pone.0182032 (PMC5531504; doi:10.1371/journal.pone.0182032)
Supplement: S3 Table — (PDF) [file pone.0182032.s003.pdf]

S3 Table. Specific up-regulated genes in mouse embryonic stem cells exposed to p-cresol (Top 30)

Refseq	Exposure/Control
NM_025669	71023
NM_001291482	27060
NM_028118	17294
NM_001253804	10330
NM_001290794	9417
NM_001290986	9417
NM_001276455	8853
NM_133879	7471
NM_001110309	7197
NM_007547	6674
NM_001167864	6495
NR_027375	6228
NM_001013368	6204
NM_001172136	6188
NM_008578	6038
NM_009685	5813
NM_013512	5323
NM_178734	5184
NM_009952	5156
NM_001102611	5056
NM_145415	4969
NM_001177607	4930
NM_001082536	4744
NM_001301305	4570
NM_001253736	4562
NM_001289440	4433
NM_001297607	4378
NR_028079	4378
NM_144917	4302
NM_172778	4191
