# Supplementary material for: Identification of RNA biomarkers for chemical safety screening in mouse embryonic stem cells using RNA deep sequencing analysis
Source: PLoS One. 2017 Jul 27;12(7):e0182032. doi: 10.1371/journal.pone.0182032 (PMC5531504; doi:10.1371/journal.pone.0182032)
Supplement: S4 Table — (PDF) [file pone.0182032.s004.pdf]

S4 Table. Specific up-regulated genes in mouse embryonic stem cells exposed to p-dichlorobenzene (Top 30)

Refseq	Exposure/Control
NM_001164745	11178
NM_001110309	6885
NM_027949	6586
NM_178734	6391
NM_001290502	6181
NR_033527	5796
NM_001302206	5425
NM_021514	5414
NM_001172136	5384
NM_010918	5207
NM_001252520	5174
NM_009685	5056
NM_001285498	5001
NM_172268	4898
NM_183308	4431
NM_172778	4393
NM_029612	4371
NM_001168679	4315
NM_139297	4072
NM_024251	4067
NM_001039533	3987
NM_198620	3962
NM_001039934	3962
NM_001159365	3949
NM_011193	3770
NM_001271353	3573
NM_145382	3558
NM_146114	3536
NM_001099624	3468
NM_177574	3466
