# Supplementary material for: Identification of RNA biomarkers for chemical safety screening in mouse embryonic stem cells using RNA deep sequencing analysis
Source: PLoS One. 2017 Jul 27;12(7):e0182032. doi: 10.1371/journal.pone.0182032 (PMC5531504; doi:10.1371/journal.pone.0182032)
Supplement: S5 Table — (PDF) [file pone.0182032.s005.pdf]

S5 Table. Specific up-regulated genes in mouse embryonic stem cells exposed to phenol (Top 30)

Refseq	Exposure/Control
NM_001291482	15627
NM_145382	14154
NM_001164745	12894
NM_025669	12078
NR_027375	8832
NM_001162465	8164
NM_183308	6629
NM_001301156	6352
NM_001162973	5897
NM_001113364	5842
NM_178045	5589
NR_110962	5351
NM_011629	5235
NR_046364	4880
NM_029612	4840
NM_001252468	4796
NM_001291777	4763
NM_177387	4710
NM_001162479	4523
NM_001004185	4506
NM_030066	4483
NM_001290986	4381
NM_007465	4354
NM_001193660	4257
NM_172279	4255
NM_001167864	4161
NM_001082536	3912
NM_010589	3909
NM_013512	3904
NM_001145957	3895
