# Supplementary material for: Identification of RNA biomarkers for chemical safety screening in mouse embryonic stem cells using RNA deep sequencing analysis
Source: PLoS One. 2017 Jul 27;12(7):e0182032. doi: 10.1371/journal.pone.0182032 (PMC5531504; doi:10.1371/journal.pone.0182032)
Supplement: S6 Table — (PDF) [file pone.0182032.s006.pdf]

S6 Table. Specific up-regulated genes in mouse embryonic stem cells exposed to pyrocatechol (Top 30)

Refseq	Exposure/Control
NM_025669	50377
NM_001291482	21948
NM_001271360	21262
NM_172372	11361
NM_001252520	9891
NM_001290794	9167
NM_001004185	9018
NM_001102611	8749
NM_198884	8092
NM_144517	8050
NM_001164626	7992
NM_001110309	7516
NM_013512	7376
NM_001276485	7097
NM_026604	7074
NM_001286483	7023
NM_027886	6909
NM_001163702	6736
NM_011896	6612
NM_011629	6605
NM_153501	6467
NM_007395	6405
NM_001013368	6363
NM_001276455	6239
NM_001177968	6197
NM_001199967	6183
NM_001289440	6108
NM_001285498	5952
NM_053124	5691
NM_025674	5633
