# Supplementary material for: Identification of RNA biomarkers for chemical safety screening in mouse embryonic stem cells using RNA deep sequencing analysis
Source: PLoS One. 2017 Jul 27;12(7):e0182032. doi: 10.1371/journal.pone.0182032 (PMC5531504; doi:10.1371/journal.pone.0182032)
Supplement: S7 Table — (PDF) [file pone.0182032.s007.pdf]

S7 Table. Specific up-regulated genes in mouse embryonic stem cells exposed to tri-n-butyl phosphate (Top 30)

Refseq	Exposure/Control
NM_025669	90352
NM_001166413	19999
NM_139297	11175
NM_001276455	8832
NM_028118	7802
NM_001110309	7577
NM_001301156	7516
NM_001033326	6379
NM_175472	6360
NM_133879	6160
NM_001079849	6112
NM_001083329	6095
NR_027375	5970
NR_033736	5814
NM_001083887	5792
NM_001165982	5663
NM_009601	5592
NM_001085472	5469
NM_183308	5368
NM_001099624	5200
NM_178734	5005
NM_001172136	4998
NM_145151	4915
NM_001024922	4806
NM_001167864	4796
NM_177574	4718
NM_001177607	4676
NM_001013368	4602
NM_175511	4581
NM_009769	4488
