# Supplementary material for: Identification of RNA biomarkers for chemical safety screening in mouse embryonic stem cells using RNA deep sequencing analysis
Source: PLoS One. 2017 Jul 27;12(7):e0182032. doi: 10.1371/journal.pone.0182032 (PMC5531504; doi:10.1371/journal.pone.0182032)
Supplement: S8 Table — (PDF) [file pone.0182032.s008.pdf]

S8 Table. Specific up-regulated genes in mouse embryonic stem cells exposed to trichloroethylene (Top 30)

Refseq	Exposure/Control
NM_001163684	11111
NM_001177607	8851
NM_001243968	8294
NM_001193660	8249
NM_001285498	8049
NR_027375	7685
NM_001276493	6744
NM_018827	6467
NM_001162973	5924
NM_001287015	5726
NM_001290502	5496
NR_035500	5447
NM_001252574	5358
NM_001163640	5069
NM_145151	4993
NM_134161	4835
NM_001301641	4640
NR_038126	4556
NM_172372	4497
NM_001163185	4417
NR_110964	4404
NM_001101535	4337
NM_001291777	4030
NM_001204202	3836
NM_001193619	3797
NM_153578	3780
NM_001253736	3753
NM_011629	3710
NM_001136079	3541
NM_024251	3526
