# Supplementary material for: Identification of RNA biomarkers for chemical safety screening in mouse embryonic stem cells using RNA deep sequencing analysis
Source: PLoS One. 2017 Jul 27;12(7):e0182032. doi: 10.1371/journal.pone.0182032 (PMC5531504; doi:10.1371/journal.pone.0182032)
Supplement: S9 Table — (PDF) [file pone.0182032.s009.pdf]

S9 Table. Specific down-regulated genes in mouse embryonic stem cells exposed to bis(2-ethylhexyl)phthalate (Top 30)

Refseq	Exposure/Control
NM_001277310	0.000059
NM_058214	0.000074
NM_001040691	0.000088
NM_009146	0.000096
NM_007434	0.000112
NM_001164195	0.000153
NM_153489	0.000164
NM_011242	0.000167
NM_001166648	0.000168
NM_021467	0.000168
NM_138309	0.000173
NM_027585	0.000175
NM_145503	0.000177
NM_001272070	0.000186
NM_026035	0.000187
NM_028705	0.000190
NM_144892	0.000193
NM_001198833	0.000195
NM_007647	0.000198
NM_001146298	0.000203
NM_001285783	0.000205
NM_199465	0.000206
NM_001163553	0.000208
NM_001005370	0.000210
NM_175151	0.000225
NM_001127169	0.000225
NM_001177469	0.000226
NM_001177372	0.000232
NM_001285988	0.000233
NM_144932	0.000233
