# Supplementary material for: Identification of RNA biomarkers for chemical safety screening in mouse embryonic stem cells using RNA deep sequencing analysis
Source: PLoS One. 2017 Jul 27;12(7):e0182032. doi: 10.1371/journal.pone.0182032 (PMC5531504; doi:10.1371/journal.pone.0182032)
Supplement: S10 Table — (PDF) [file pone.0182032.s010.pdf]

S10 Table. Specific down-regulated genes in mouse embryonic stem cells exposed to chloroform (Top 30)

Refseq	Exposure/Control
NM_001205334	0.000076
NM_146248	0.000136
NM_001256522	0.000165
NM_001098231	0.000186
NM_016846	0.000201
NM_152808	0.000203
NM_029128	0.000207
NM_199465	0.000207
NM_001163553	0.000209
NM_178698	0.000216
NR_102360	0.000216
NM_001272076	0.000220
NM_001161370	0.000224
NM_001291036	0.000240
NM_001013365	0.000252
NM_145978	0.000256
NM_146188	0.000257
NM_172911	0.000263
NM_177338	0.000274
NM_001277170	0.000274
NM_001276335	0.000286
NM_181071	0.000308
NM_172772	0.000309
NM_001302951	0.000317
NM_172570	0.000318
NM_001271915	0.000327
NM_013866	0.000327
NM_001045513	0.000330
NM_001040400	0.000341
NM_001039387	0.000344
