# Supplementary material for: Identification of RNA biomarkers for chemical safety screening in mouse embryonic stem cells using RNA deep sequencing analysis
Source: PLoS One. 2017 Jul 27;12(7):e0182032. doi: 10.1371/journal.pone.0182032 (PMC5531504; doi:10.1371/journal.pone.0182032)
Supplement: S11 Table — (PDF) [file pone.0182032.s011.pdf]

S11 Table. Specific down-regulated genes in mouse embryonic stem cells exposed to p-cresol (Top 30)

Refseq	Exposure/Control
NM_001045553	0.000054
NM_058214	0.000074
NM_019989	0.000113
NM_001033528	0.000120
NM_001001321	0.000133
NM_010188	0.000147
NM_001164195	0.000154
NM_001159510	0.000159
NM_001193661	0.000159
NM_001166648	0.000168
NM_021467	0.000169
NM_032460	0.000170
NM_001033284	0.000170
NM_001037754	0.000206
NR_102360	0.000215
NM_001039137	0.000220
NR_037964	0.000225
NM_146221	0.000236
NM_001205053	0.000237
NM_001285431	0.000240
NM_011618	0.000250
NM_175335	0.000250
NM_145978	0.000255
NM_001286824	0.000256
NM_010059	0.000257
NM_011461	0.000258
NM_001081173	0.000277
NM_001276335	0.000285
NM_146135	0.000312
NM_133789	0.000316
