# Supplementary material for: Identification of RNA biomarkers for chemical safety screening in mouse embryonic stem cells using RNA deep sequencing analysis
Source: PLoS One. 2017 Jul 27;12(7):e0182032. doi: 10.1371/journal.pone.0182032 (PMC5531504; doi:10.1371/journal.pone.0182032)
Supplement: S12 Table — (PDF) [file pone.0182032.s012.pdf]

S12 Table. Specific down-regulated genes in mouse embryonic stem cells exposed to p-dichlorobenzene (Top 30)

Refseq	Exposure/Control
NR_028297	0.000054
NM_001161338	0.000074
NM_001045553	0.000113
NM_058214	0.000120
NM_146105	0.000133
NM_001033528	0.000147
NM_177460	0.000154
NM_001178058	0.000159
NM_001302205	0.000159
NM_018812	0.000168
NM_001293767	0.000169
NM_001289581	0.000170
NM_001109748	0.000170
NM_010188	0.000206
NM_020507	0.000215
NM_001159510	0.000220
NM_001166648	0.000225
NM_001025311	0.000236
NM_001032378	0.000237
NM_026035	0.000240
NM_028705	0.000250
NM_198298	0.000250
NM_013869	0.000255
NR_027396	0.000256
NM_001282024	0.000257
NM_024479	0.000258
NM_009538	0.000277
NM_199027	0.000285
NR_037590	0.000312
NM_021326	0.000316
