# Supplementary material for: Identification of RNA biomarkers for chemical safety screening in mouse embryonic stem cells using RNA deep sequencing analysis
Source: PLoS One. 2017 Jul 27;12(7):e0182032. doi: 10.1371/journal.pone.0182032 (PMC5531504; doi:10.1371/journal.pone.0182032)
Supplement: S13 Table — (PDF) [file pone.0182032.s013.pdf]

S13 Table. Specific down-regulated genes in mouse embryonic stem cells exposed to phenol (Top 30)

Refseq	Exposure/Control
NM_019989	0.000113
NM_030255	0.000115
NM_001159571	0.000127
NM_001081373	0.000134
NM_001290745	0.000136
NM_001159498	0.000150
NM_011075	0.000160
NM_153489	0.000164
NM_134029	0.000167
NM_001166648	0.000169
NM_177352	0.000174
NM_026035	0.000188
NM_001037754	0.000207
NM_001163553	0.000209
NM_001005370	0.000211
NM_133687	0.000215
NM_001289709	0.000215
NR_037964	0.000225
NM_146221	0.000236
NM_001285431	0.000241
NM_173417	0.000247
NM_175335	0.000251
NM_001190322	0.000255
NM_146188	0.000257
NM_001205053	0.000285
NM_001301155	0.000290
NM_001286986	0.000300
NR_105022	0.000302
NM_015775	0.000310
NM_001304831	0.000312
