# Supplementary material for: Identification of RNA biomarkers for chemical safety screening in mouse embryonic stem cells using RNA deep sequencing analysis
Source: PLoS One. 2017 Jul 27;12(7):e0182032. doi: 10.1371/journal.pone.0182032 (PMC5531504; doi:10.1371/journal.pone.0182032)
Supplement: S14 Table — (PDF) [file pone.0182032.s014.pdf]

S14 Table. Specific down-regulated genes in mouse embryonic stem cells exposed to pyrocatechol (Top 30)

Refseq	Exposure/Control
NM_001177595	0.000092
NM_009595	0.000110
NM_001102423	0.000113
NM_177602	0.000116
NM_018812	0.000133
NM_001286009	0.000142
NR_073442	0.000152
NM_001159696	0.000157
NM_152808	0.000158
NM_001291052	0.000163
NM_001166648	0.000168
NM_001098231	0.000185
NM_176860	0.000205
NM_199465	0.000206
NM_001163553	0.000208
NM_177054	0.000212
NR_102360	0.000215
NM_001177469	0.000226
NM_001035509	0.000234
NM_001110148	0.000235
NM_001205053	0.000236
NM_139232	0.000239
NM_001285431	0.000240
NR_027651	0.000243
NM_030000	0.000245
NM_011618	0.000249
NM_145978	0.000255
NM_015819	0.000257
NR_033185	0.000263
NM_013508	0.000274
