# Supplementary material for: Identification of RNA biomarkers for chemical safety screening in mouse embryonic stem cells using RNA deep sequencing analysis
Source: PLoS One. 2017 Jul 27;12(7):e0182032. doi: 10.1371/journal.pone.0182032 (PMC5531504; doi:10.1371/journal.pone.0182032)
Supplement: S15 Table — (PDF) [file pone.0182032.s015.pdf]

S15 Table. Specific down-regulated genes in mouse embryonic stem cells exposed to tri-n-butyl phosphate (Top 30)

Refseq	Exposure/Control
NM_028027	0.000047
NM_001045553	0.000054
NM_001252253	0.000117
NM_025530	0.000143
NM_001198835	0.000150
NM_001289726	0.000152
NM_001111063	0.000160
NM_026486	0.000169
NM_023536	0.000171
NM_001098231	0.000186
NM_026035	0.000188
NM_028705	0.000191
NM_001037754	0.000207
NM_009538	0.000221
NM_001177469	0.000227
NM_001285431	0.000241
NR_027651	0.000244
NM_001282065	0.000244
NR_027876	0.000245
NM_011618	0.000251
NM_008087	0.000251
NM_001291443	0.000255
NM_145978	0.000256
NM_029601	0.000263
NR_033185	0.000264
NM_001252384	0.000285
NM_008954	0.000285
NM_001302086	0.000296
NM_001159620	0.000296
NM_017379	0.000302
