# Supplementary material for: Identification of RNA biomarkers for chemical safety screening in mouse embryonic stem cells using RNA deep sequencing analysis
Source: PLoS One. 2017 Jul 27;12(7):e0182032. doi: 10.1371/journal.pone.0182032 (PMC5531504; doi:10.1371/journal.pone.0182032)
Supplement: S16 Table — (PDF) [file pone.0182032.s016.pdf]

S16 Table. Specific down-regulated genes in mouse embryonic stem cells exposed to trichloroethylene (Top 30)

Refseq	Exposure/Control
NM_026489	0.000058
NM_001165986	0.000069
NM_001111063	0.000077
NM_001081368	0.000086
NM_001161338	0.000107
NM_001289599	0.000110
NM_010783	0.000119
NM_013846	0.000129
NM_001081373	0.000133
NM_146248	0.000135
NM_001289581	0.000138
NM_001252470	0.000148
NM_178628	0.000155
NM_010435	0.000160
NM_001166648	0.000168
NM_001033284	0.000170
NM_001285433	0.000174
NM_001272070	0.000186
NM_001285783	0.000204
NM_001282024	0.000205
NM_011600	0.000205
NM_001163553	0.000207
NM_001127169	0.000225
NM_016875	0.000237
NM_001291449	0.000237
NM_001276285	0.000238
NM_001291036	0.000239
NM_144842	0.000248
NM_175335	0.000249
NM_001291443	0.000253
